# The Activity of Special Continuity Care Units in the City of Florence During the COVID-19 Pandemic

**DOI:** 10.3389/ijph.2023.1606338

**Published:** 2023-10-06

**Authors:** Chiara Milani, Primo Buscemi, Beatrice Velpini, Giulia Naldini, Claudia Cosma, Francesco Giannuzzi, Giulia Ionita, Pietro Monaci, Ruggero Landi, Irene Pontalti, Lorenzo Baggiani, Marco Nerattini, Chiara Lorini, Guglielmo Bonaccorsi

**Affiliations:** ^1^ Department of Health Science, University of Florence, Florence, Italy; ^2^ School of Specialization in Hygiene and Preventive Medicine, University of Florence, Florence, Italy; ^3^ Department of District Health Care Network, Azienda USL Toscana Centro, Florence, Italy; ^4^ Ex-Special Continuity Care Units, Azienda USL Toscana Centro, Florence, Italy; ^5^ Health Society of Florence, Florence, Italy

**Keywords:** COVID-19, primary care, public health, home care services, multiprofessional team

## Abstract

**Objectives:** Worldwide, countries adopted different strategies in primary care (PC) to cope with the COVID-19 pandemic. This study aims to describe and evaluate the functions and activity load of a specific PC organizational model called “Special Continuity Care Units” (SCCU) in Florence, Italy, and to investigate the characteristics of the COVID-19 patients assisted by the service.

**Methods:** The retrospective cross-sectional design used daily updated reports by SCCU team members to evaluate the activity load. The retrospective cohort study analyzed data of the demographics, clinical characteristics, and process outcomes of patients assisted during the second pandemic wave.

**Results:** The analysis shows how the service activity load changed along with the epidemiological trend. Regarding people assisted by the SCCU, the median follow-up duration of symptoms was 6 days; male gender and being symptomatic were predictors of hospitalization.

**Conclusion:** Some key characteristics can be described as indispensable in PC services facing health emergencies: model flexibility, the availability of resources, networking among services to enhance coordination and resource optimization, and close collaboration with general practitioners.

## Introduction

### Primary Healthcare and COVID-19

On 11 March 2020, the World Health Organization (WHO) declared COVID-19 a pandemic [1]. COVID-19 most severely affected vulnerable, elderly people, those with comorbidities [2–5], and individuals living in care homes and other community settings [6].

The sudden increase in COVID-19 cases resulted in a rising workload for hospitals and primary care (PC) services. In this context, the need to reorganize primary care services became a priority and was placed at the center of public health debate [7, 8]. Indeed, primary healthcare (PHC) has always represented an essential element of the global response to health emergencies and risk management [9–11]. In fact, it plays a specific role in the management and containment during the initial response, triage, early diagnosis, treatment, and surveillance, as well as in reducing the demand for hospital services [12]: supporting home care is crucial when healthcare facilities are under pressure because of shortages of hospital beds [13].

Different countries defined specific strategies to cope with COVID-19 within the context of PC services. Recent studies compared the lessons learned in PC services during previous infectious disease epidemics, highlighting the relevance of strengthening these services and improving their collaboration and integration in pandemic response [11, 14, 15]. Kumpunen et al. identified three prevalent PHC delivery models applied in response to the pandemic across Europe: multi-disciplinary PC teams and public health functions of surveillance, planning, and evaluation being coordinated in their contributions to the emergency response; PHC providers prioritizing vulnerable patients; and digital solutions enhancing the effectiveness of the PHC response [16]. Other specific solutions included dedicated specialist hospitals and outpatient clinics aimed at assessing severity and arranging for hospitalization if needed [17], such as “fever clinics” established in China or Germany to identify SARS-CoV-2 infections [18, 19]. In China, “shelter hospitals” were also established in a short time as another attempt to respond to the pandemic [20].

### Primary Care and COVID-19 in Italy

Since the global outbreak of SARS-CoV-2 infections, Italy has been among the countries with the highest burden of disease. By December 2022, the total number of COVID-19 cases in Italy was more than 25 million, with 182,000 deaths. According to the information provided by the Italian National Health Institute (ISS, Istituto Superiore di Sanità), the first pandemic wave occurred between February and May 2020, the second between late summer of 2020 and January 2021 with a peak in October 2020, the third between February and May 2021. A fourth rise in COVID-19 cases was observed between June and October 2021, while the Omicron variant was detected for the first time on 27 November 2021 [21].

The Italian National Health Service (NHS) provides PC services by means of the Local Health Districts (LHD), in which healthcare and social services are given jointly.

To forefront the COVID-19 pandemic, the Italian government improved PC services and set up within LHDs medical teams called “Special Continuity Care Units” (SCCU) (*Unità Speciali di Continuità Assistenziale—USCA*). SCCUs supported general practitioners (GPs) in home management of suspected or confirmed COVID-19 patients, with a ratio of 1–50,000 inhabitants, 7 days a week, from 8.00 a.m. to 8.00 p.m. They were activated by GPs or by other health and social services—for example emergency services or hospitals—which reported to SCCUs the name, address, and clinical conditions of the patient after a telephone triage. Each Italian region managed SCCUs differently, entrusting them with different functions and organizational models.

### Aim

The primary aim of our study is to evaluate the functions and activity load of the Florence SCCU—a PC model adopted to support home management of COVID-19 patients—in the LDH of Florence (Tuscany, Italy), evolving with the epidemiological trend from 1 June 2020 to 30 March 2022.

Moreover, the study investigates the demographic and clinical characteristics of COVID-19 patients assisted by the SCCU during the second pandemic wave in Italy that heavily affected the population in the pre-vaccination period and during which the provision of additional diagnostic instruments enhanced the SCCU home management of COVID-19 patients. Considering this sample, it also analyzes the outcomes of processes to gain a better understanding of the organizational elements that can be applied even after the end of the pandemic emergency.

## Methods

### Study Design and Setting

This single-center study consisted of two parts. In the first, an investigation of the activities of the SCCU from 1 June 2020 to 30 March 2022 took place with a retrospective cross-sectional design. In the second, an analysis of demographic and clinical data and the health outcomes of patients cared for by the SCCU in Florence from 1 November 2020 to 15 December 2020 was performed with a retrospective cohort design. The study was realized according to the principles of the Helsinki Declaration and approved by the local ethics committee (Register number: 20609_oss).

### Context

The activity of the SCCU in Florence started at the beginning of April 2020 and served a population of 361,619 people (figure reported as of 31 December 2021) [22]. The local SCCU model consisted of primary care teams of physicians and nurses working together, with the support of other healthcare professionals when appropriate (Table 1).

**TABLE 1:** Health and social professionals (physicians, nurses, assistants/social-health operators, administrative assistant/staff, and primary care medical managers and residents in public health) composing the services of the Florence SCCU and their activities (Italy, 2023).

Physicians	Clinical management of SARS-CoV-2 infection (clinical evaluation and therapy)
	Telephone follow-up
	Work planning and management
	Report management
	Opening, closing, and filing of medical history
	Performing SARS-CoV-2 nasopharyngeal swabs, checking the result, and communication with the patient
	Cooperation with GPs and clinical reports
	Reporting of daily activity
	Performing and interpretating first-line diagnostics (electrocardiogram, hemogasanalysis, and lung ultrasound) COVID-19 vaccinations
Nurses	Nursing (bladder and venous catheterization, patient mobilization, phleboclysis, and dressings)
	Home blood sampling in COVID-19 patients
	Performing SARS-CoV-2 nasopharyngeal swabs
	Internal logistics activities
	COVID-19 vaccinations
Assistants/social-health operators	Collaboration with nurses in all the activities listed above
Administrative assistant/staff	Administrative support in activity planning
Primary care medical manager and residents in public health	Activity planning
	Activity monitoring
	Monitoring the COVID-19 disease spread in care settings, such as “COVID-19 hotels” and nursing homes
	Networking the social-health services in the primary health district to warrant the continuity of care through different settings

The Florence SCCU performed the following activities:

- the clinical evaluation of COVID-19 patients at home and in cooperation with GPs;
- medical prescriptions;
- home administration of medications and oxygen therapy;
- execution of SARS-CoV-2 nasopharyngeal swab in COVID-19 patients;
- first-line diagnostic assessment (blood sampling, electrocardiogram, hemogasanalysis, bedside chest X-rays, and lung ultrasounds with portable radiological support);
- percutaneous gastrostomy endoscopic (PEG) replacement in COVID-19 patients;
- telephone follow-up;
- clinical evaluation of patients and follow-up in “COVID-19 hotels”; clinical evaluation of patients in nursing homes; clinical monitoring in cooperation with GPs and the mobile geriatric assessment team;
- monitoring and control of healthcare facilities with COVID-19 outbreaks (nursing homes, structures for marginalized and socially excluded people, etc.);
- COVID-19 vaccinations at home for frail people;
- COVID-19 vaccinations at residential facilities and towards marginalized and socially excluded people;
- cooperation with the Florentine Agency for Hospital-Territory Continuity of Care (AHTCC) for: multidisciplinary evaluation of complex patients, when reported by the hospital physicians; home management planning; supporting patients remaining at home in cooperation with the public prevention service.

The Florence SCCU managed COVID-19 cases in patients at home and in specific residential facilities, such as nursing homes and “COVID-19 hotels,” where people unable to be isolated at home recovered.

In particular, the Florence SCCU took care of:

- COVID-19 patients with mild respiratory symptoms, aged >70 years, and/or with comorbidities or at increased risk of mortality (fever >37.5°C; mild or moderate cough or progressive coughing);
- COVID-19 patients with the previously described symptoms who did not require hospitalization or had been discharged from hospital;
- COVID-19 patients and patients with suspected SARS-CoV-2 infection with respiratory symptoms and persistent (>4 days) fever or asthenia and cough.

### Data Collection

Regarding the retrospective cross-sectional study, data from home clinical evaluations and nasopharyngeal swabs were collected using a daily updated report by the SCCU team members. The study focused on the activities characterizing the home management of COVID-19 patients (which was the focus of the second part of the study) throughout the entire period of the Florence SCCU activity.

For the retrospective cohort study, data extracted by IT software used to record patient data (Caribel/Aster, Tuscany, Italy) were integrated with data collected from paper-based medical records. Data on demographics (gender, age, and citizenship), clinical data (symptoms related to COVID-19, patient’s autonomy, and comorbidities for chronic diseases), and data on process indicators and outcomes (origin of the report, previous hospitalization, admission to hospital or low intensity care settings, and days from the onset of symptoms to presentation) were collected. The COVID-19 status in terms of confirmed or suspected cases was recorded, including real time polymerase chain reaction (RT-PCR) tests with date of execution and result.

The inclusion criteria were the following: patients aged 18 years and over, living in three out of the five neighborhoods (Q1, Q4, and Q5) of Florence (drawn at random and corresponding to about 65% of residents in Florence), suspected or confirmed COVID-19 cases (positive RT-PCR test on nasopharyngeal swab), and with at least one home clinical evaluation from 1 November 2020 to 15 December 2020. People in residential facilities and COVID-19 hotels were excluded. The follow-up ended with hospitalization, the transition to a different care setting, or the completion of the treatment program.

### Statistical Analysis

According to the epidemiological trend, in the retrospective cross-sectional design, five epidemic periods were considered: the first period was from 1 June to 30 September 2020; the second period was from 1 October 2020 to 31 January 2021; the third period was from 1 February 2021 to 31 May 2021; the fourth period was from 1 June to 30 September 2021; and the fifth period was from 1 October 2021 to 30 March 2022. Table 2 provides a description of their main characteristics.

**TABLE 2:** Main characteristics of the five epidemic periods analyzed (Italy, 2023).

Period	Date range	COVID-19 cases incidence	COVID-19 mass vaccination campaign
First	1 June–30 September 2020	Low	Before starting
Second	1 October 2020–31 January 2021	High	Before starting and just started
Third	1 February 2021–31 May 2021	High	Ongoing
Fourth	1 June–30 September 2021	Low	Ongoing
Fifth	1 October 2021–30 March 2022	High	Ongoing

The 7 days moving averages of the daily number of home clinical evaluations and nasopharyngeal swabs performed during the considered periods were calculated. The peak values of home clinical evaluations or nasopharyngeal swabs for each period were computed as the highest 7 days moving average value. For every considered period, the absolute number of home clinical evaluations and swabs performed during the 2 weeks around the peak value of home clinical evaluations or nasopharyngeal swabs was recorded. Relative percent difference was calculated as the value for home clinical evaluations and nasopharyngeal swabs from the considered period minus the same indicator value from the comparison period, divided by the same indicator value from the comparison period. The following comparisons were performed: the third period vs. the second period; the fifth period vs. the second period; the fifth period vs. the third period; and the fourth period vs. the first period. For the first period, no data on nasopharyngeal swabs were available.

The retrospective cohort study included descriptive analysis of patients’ characteristics. For continuous non-normally distributed variables, the median and interquartile range (IQR) are reported. Categorical variables are expressed as percentages. The positivity rate in nasopharyngeal swabs performed by the Florence SCCU was calculated by dividing the number of positive tests by the total number of executed tests, and multiplying by 100.

A Cox regression analysis was performed to investigate the association between the baseline characteristics of COVID-19 patients (*N* = 544) and the admission to hospital or low intensity setting of care. Two Cox regression models were used. The first model included the following covariates: gender, age group, citizenship, COVID-19 symptoms at presentation, coexistence of chronic diseases, and previous hospitalization. The final model included the following variables: gender, age group, and COVID-19 symptoms at presentation. The variables included in the final model were selected using the backward stepwise selection method. The assumption of proportional hazard was checked using the Schoenfeld residual test (Supplementary Tables S1A, S1B; Supplementary Figures S1A, S1B).

For all the analyses, a significance threshold of *p* < 0.05 was adopted.

The statistical analysis was performed using the R software (version 4.1.0).

## Results

### SCCU Performances (Retrospective Cross-Sectional Design)

The highest number of home clinical evaluations was performed during the third period (*N* = 864, peak value of 7 days moving average: 59.6). An increase of 6.4% in home clinical evaluations was observed (third period: *N* = 864; second period: *N* = 812) compared with the second period. In the fourth period, home clinical evaluations almost doubled compared with the home medical visits recorded in the first period (relative difference: 92.5%; fourth period: *N* = 258; first period: *N* = 134). During the fifth period, a decrease in home clinical evaluations of 30.5% and 34.7% was observed compared, respectively, to the second period and the third period (fifth period: *N* = 564; second period: *N* = 812; third period: *N* = 864). The activities of the Florence SCCU throughout the different periods of study are shown in Supplementary Tables S2A, S2B.

The largest number of nasopharyngeal swabs was administered during the second period (*N* = 622, with a peak value of 7 days moving average: 45.4). During the third period, a decrease of 57.9% of swabs administered was observed compared with the second period (second period: *N* = 622; third period: *N* = 262). In the fifth period (*N* = 303), the number of nasopharyngeal swabs was lower compared with the second period (−51.3%), but higher compared with the third period (15.6%).

Tables 3, 4 show the comparison of the Florence SCCU home clinical evaluations and swabs during the defined period.

**TABLE 3:** Comparison of the Florence SCCU home clinical evaluations (difference and relative difference) during the five epidemic periods (third period vs. second period, fifth period vs. second period, fifth period vs. third period, and fourth period vs. first period) (Italy, 2023).

Epidemiological period	Analyzed period (date range)	Difference (*N*)	Relative difference (%)
Comparison of third period with second period	01/04/2021–15/04/2021 vs. 30/10/2020–13/11/2020	52	6.4
Comparison of fourth period with first period	18/08/2021–01/09/2021 vs. 22/08/2020–05/09/2020	124	92.5
Comparison of fifth period with second period	11/01/2022–25/01/2022 vs. 30/10/2020–13/11/2020	−248	−30.5
Comparison of fifth period with third period	11/01/2022–25/01/2022 vs. 01/04/2021–15/04/2021	−300	−34.7

**TABLE 4:** Comparison of the Florence SCCU swabs during the five epidemic periods (difference, and relative difference) (Italy, 2023).

Epidemiological period	Analyzed period (date range)	Difference (*N*)	Relative difference (%)
Comparison of third period with second period	01/04/2021–15/04/2021 vs. 31/10/2020–14/11/2020	−360	−57.9
Comparison of fifth period with second period	26/12/2021–09/01/2022 vs. 31/10/2020–14/11/2020	−319	−51.3
Comparison of fifth period with third period	26/12/2021–09/01/2022 vs. 01/04/2021–15/04/2021	41	15.6

Characteristics and outcomes of patients in home setting during the second pandemic wave in Italy—second period (the retrospective cohort study).

From 1 November to 15 December 2020, a total of 769 patients were referred to the SCCU service: 53.1% were females (*N* = 408); the median age was 61.5 years (IQR 45.0–77.3); 12.4% were non-EU citizens (*N* = 95); most of the patients (84.8%, *N* = 652) were reported to the SCCU service from GPs, 10.7% (*N* = 82) from the Agency for Hospital-Territory Continuity of Care (AHTCC), and 4.6% (*N* = 35) from other reporting services (i.e., emergency services, hospital, and public hygiene facilities). At the time of being reported to the SCCU, 67.4% of the patients (*N* = 518) represented suspected COVID-19 cases and 32.6% (*N* = 250) were confirmed COVID-19 cases. A total of 4.6% of patients (*N* = 35) were discharged from a hospital facility where they had been admitted for COVID-19 disease. Furthermore, 80.1% of patients (*N* = 613) suffered from COVID-19 related symptoms, 55.1% (*N* = 424) had at least one chronic disease, and 14.2% (*N* = 108) were severely dependent.

Among suspected cases, 506 nasopharyngeal swabs were performed: 58.1% (*N* = 294) were positive for COVID-19, 37.7% (*N* = 191) negative, 1.8% (*N* = 9) low-blood positive, and 2.4% (*N* = 12) yielded inconclusive results. The positivity rate was higher in males than in females (61.8% vs. 55.8%) and in non-EU citizens than in EU citizens than in Italian patients (70.6% vs. 56.8%).

Overall, during the observation period, the SCCU took care of 544 confirmed COVID-19 patients (70.8% of patients). The median follow-up duration in confirmed COVID-19 cases was 6 days (IQR: 2–15). A total of 89.3% of confirmed COVID-19 patients (*N* = 486) were referred back to the GP after having completed the treatment program; 9.7% were hospitalized (*N* = 53) and five patients were admitted to a low intensity setting of care. Furthermore, 80.8% (*N* = 437) of confirmed COVID-19 patients presented COVID-19-related symptoms, 53.9% had at least one chronic disease, and 12% (*N* = 65) were severely dependent. Table 5 shows the sample characteristics.

**TABLE 5:** Demographic characteristics, clinical characteristics, and outcomes of patients cared for by the SCCU (whole sample and lab-confirmed COVID-19 patients), *N* = 769 (Italy, 2023).

Sample			Total patients (*N* = 769)	COVID-19 positive patients (*N* = 544)
Demographic characteristics	Age (mean)		61.5 (45.0–77.3)	59.9 (45.2–75.1)
	Sex	Female	408 (53.1%)	283 (52.0%)
		Male	361 (46.9%)	261 (48.0%)
	Citizenship	UE	674 (87.6%)	470 (86.4%)
		EU and non-EU	95 (12.4%)	74 (13.6%)
Clinical characteristics	Symptomatic patients		613 (79.7%)	437 (80.3%)
	Chronic diseases (at least one)		424 (55.1%)	293 (53.9%)
	Patients severely dependent in daily activities		70 (11.6%)	61 (13%)
Outcomes	Completion of treatment program		694 (90.2%)	486 (89.3%)
	Hospitalization		68 (8.8%)	53 (9.7%)
	Admission to a low care intensity setting		5 (0.7%)	5 (0.9%)
	Deaths		2 (0.3%)	0 (0%)

According to the Cox regression analysis, the following predictors were significantly associated with hospitalization or admission in low intensity setting of care: male gender (HR = 1.9, 95% CI: 1.1–3.2, *p* = 0.02), age group 65–85 years vs. <65 years (HR = 2.5, 95% CI: 1.4–4.4, *p* = 0.002), and being symptomatic at the first clinical evaluation (HR = 2.7, 95% CI = 1.0–6.7, *p* = 0.03). The results of the multiple Cox regression analysis are shown in Table 6.

**TABLE 6:** Hazard ratio (HR) of being admitted to hospital or a low intensity setting of care for COVID-19 patients (*N* = 544) from a multiple Cox regression analysis (final model) (Italy, 2023).

Characteristics		Hazard ratio	95% CI	*p*-value
Gender	F	—	—	—
	M	1.922	1.123; 3.288	0.017
Age group	<65 years	—	—	—
	65–85 years	2.468	1.384; 4.400	0.02
	>85 years	2.002	0.834; 4.806	0.12
COVID-19 symptoms at presentation	No	—	—	—
	Yes	2.695	1.077; 6.745	0.034

The results were adjusted for gender, age group, and symptom status at presentation.

## Discussion

This study describes the evolving organizational model of the SCCU in the LHD of Florence, which has allowed the reorganization of some specific COVID-19 care activities in non-hospital settings. The analysis showed how the service activity load changed with the epidemiological trend from 1 June 2020 to 30 March 2022. Regarding the antigen test capacity, the results showed that the third period recorded a reduction of 57.9% in the number of nasopharyngeal swabs performed by the SCCU, compared with the second period. It was probably due to the organizational context, particularly the widespread self-testing by the patients in pharmacies during the third wave, that may have affected the likelihood of having a positive test when done by the SCCU team [23]. Accordingly, the results from the retrospective cohort study showed a high number of suspected COVID-19 patients (67.4%) referred to the SCCU during the second period, when self-testing was not widespread among the population. Moreover, the number of home clinical evaluations increased by 6.4% during the third period compared with the second, and by 92.5% during the fourth period compared with the first, probably due to the easing of restrictions and to the spread of Delta variant of concern. The fifth period, characterized by the circulation of the Omicron variant, observed a reduction by 51.3% in the number of nasopharyngeal swabs compared with the second, as asymptomatic patients who did not need home clinical evaluation by the SCCU team could be referred to other services providing nasopharyngeal swabs in the LHD. Furthermore, home clinical evaluations reduced by 30.5% compared with the second period, probably due to the lower severity of symptoms and to the high vaccination coverage recorded in the LHD of Florence.

The demographic characteristics of the COVID-19 patients with positive RT-PCR assisted by the Florence SCCU in the second period are in line with the cases described at an international level [23, 24].

Considering the clinical characteristics, the proportion of asymptomatic patients (including subjects with comorbidities and/or severe dependency) in the overall sample could be explained by the following organizational elements:

• the referral of COVID-19 patients to the SCCU by GPs or other health services with a previous healthcare professional assessment;
• the composition in multi-professional teams of physicians and nurses, combining the clinical management of COVID-19 infection (clinical evaluation and therapy) with nursing assistance;
• the continuity of care ensured by means of the cooperation with the Florentine Agency for Hospital-Territory Continuity of Care addressing long- and short-term care needs.

The median follow-up duration of symptoms (6 days) was influenced by the organizational model of the SCCU, which provided home clinical evaluations, clinical reassessment, and telephone follow-up, like many other countries in Europe. In fact, a study that investigated the clinical pathway of COVID-19 patients in PC from September 2020 found that European countries patients’ follow-up in PC used phone calls, E-mail, or video consultations. In Italy, SCCUs supported GPs in this process [25].

The proportion of confirmed COVID-19 cases that underwent hospitalization (<10%) was generally lower than that recorded in other international experiences, which, however, investigated the hospitalization rate in the general population and before the beginning of the vaccination campaign [24, 26, 27]. The exception is represented by the study by Beaney et al., who reported a lower proportion of hospitalized patients but considered a longer observation period and included the start of the vaccination campaign [23]. Italian studies have shown that active monitoring by GPs and taking care of patients with home clinical evaluations is associated with a reduced risk of hospitalization in COVID-19 outpatients [28, 29].

According to the literature, different PC out-of-hospital models have been implemented in facing the COVID-19 pandemic, with some different characteristics: a model specifically dedicated to taking care of non-hospitalized COVID-19 patients, like ours, differs from a model that strengthens pre-existing services through the activity of GPs [30] or other health professionals [31, 32]; a model centered on multiprofessional teams of a physician and a nurse in close connection with emergency department and hospital bed management [33], like our model, based on an outpatient (specialist) service in PC setting[34, 35].

The above cited characteristics of different out-of-hospital models described in the literature, together with the recorded data and the results as reported from the activities of SCCUs, let us consider and stress some key features of primary care during a health emergency [11, 14, 15]:

• Flexibility of the model/service: SCCUs adjusted activities and organizational model to the evolution of healthcare needs during the different pandemic phases [7, 12].
• Flexibility in working practices: health professionals’ ability to cooperate in a changing epidemiological context, within a flexible service, experimenting with multi-professional teams which may—and perhaps should—be maintained even after the pandemic ends [7, 36].
• Resource availability in PC: it is crucial to avoid staff shortages in PC services [37]. It has happened that relocation to other settings, especially to support the shortage of professionals and overwork in hospital settings, has depleted PC services [7, 12].
• Networking: the inclusion of the service within a publicly funded LHD with a role in government, organization, and resource allocation. The networking between services enhances coordination and resource optimization [37].
• Close collaboration with GPs in terms of clinical connection before and after SCCU clinical evaluation and follow-up.
• Support in home care through the timely assessment of health needs and identification of the most appropriate treatment or assistance pathways.

These elements have been central to the organization of primary care in coping with the COVID-19 pandemic, but they should be considered in future HEs and as elements of strengthening and reorganizing PC services in the post-pandemic period, as also indicated by international organizations [7].

We are aware that our study has limitations. Data collection in the retrospective cohort design described in the study integrated paper-based medical records and data from IT health software, while data collection in the retrospective cross-sectional design included data registered in the IT software only. The lack of an integrated health information system within PC services and hospitals contributed to incomplete information regarding clinical data and outcomes. The reduced period of clinical data collection for the retrospective cohort study (second period) limited the comparison of the epidemiological phases. Activity data were mostly collected by healthcare professionals as self-reported data, which might lead to inaccuracies.

However, in spite on these limitations, to the best of our knowledge, the present study provides an assessment of a PC service aimed at the home and territorial management of confirmed or suspected COVID-19 cases in Italy. Moreover, it provides both an overview of the variation in activity load throughout the pandemic period and an in-depth focus of a specific period in terms of characteristics and outcomes.

### Conclusion

The remodeling of the SCCU activity—in line with the epidemiological trend—positively contributed to the home and territorial management of suspected and confirmed COVID-19 cases and to the sustainability of the healthcare system by reducing the pressure on emergency services and hospitals. It confirms the importance of effective primary care services in dealing with HEs. Relevant organizational elements of this PC service included the multi-professional approach, the experimentation of new collaborative working practices, the continuity of care.
