# Supplementary material for: The Activity of Special Continuity Care Units in the City of Florence During the COVID-19 Pandemic
Source: Int J Public Health. 2023 Oct 6;68:1606338. doi: 10.3389/ijph.2023.1606338 (PMC10587394; doi:10.3389/ijph.2023.1606338)
Supplement: Supplementary file 1 [file DataSheet2.docx]

**Supplementary File**

***Supplementary table 1a. Schoenfeld test for the proportional-hazards assumption: results for the first model.***

| **Variable** | **chisq** | **df** | **p** |
| --- | --- | --- | --- |
| Gender | 0.577 | 1 | 0.447 |
| Age group | 5.292 | 2 | 0.071 |
| Citizenship | 2.945 | 1 | 0.086 |
| Chronic disease | 0.465 | 1 | 0.495 |
| COVID-19 symptoms at presentation | 0.885 | 1 | 0.347 |
| Previous hospitalization | 0.011 | 1 | 0.916 |
| GLOBAL | 8.638 | 7 | 0.280 |

***Supplementary Figure 1a. Results of the Schoenfeld test: first model. The plot gives an estimate of the time-dependent coefficient beta(t). If the proportional hazards assumption holds then the true beta(t) function would be a horizontal line.***


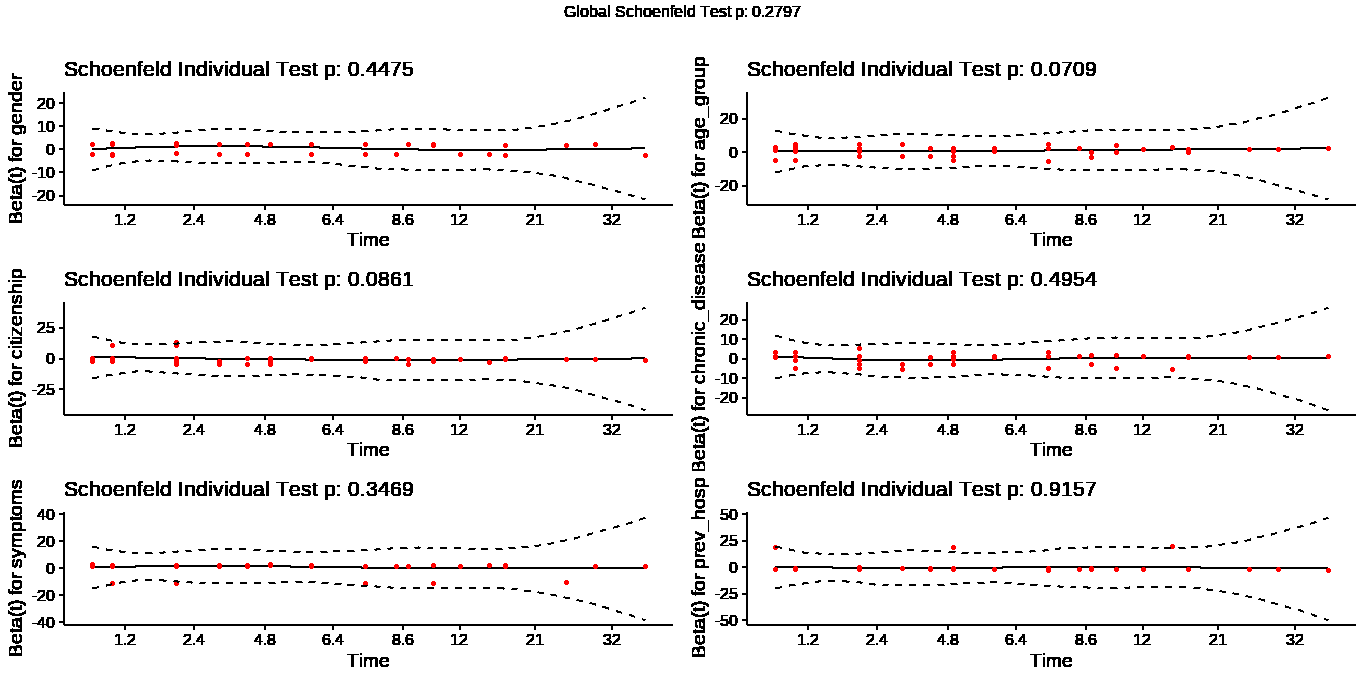


***Supplementary table 1b. Schoenfeld test for the proportional-hazards assumption: results for the final model.***

| **Variable** | **chisq** | **df** | **p** |
| --- | --- | --- | --- |
| Gender | 0.549 | 1 | 0.459 |
| Age group | 5.359 | 2 | 0.069 |
| COVID-19 symptoms at presentation | 0.869 | 1 | 0.351 |
| GLOBAL | 6.779 | 4 | 0.148 |

***Supplementary Figure 1b. Results of the Schoenfeld test: final model. The plot gives an estimate of the time-dependent coefficient beta(t). If the proportional hazards assumption holds then the true beta(t) function would be a horizontal line.***


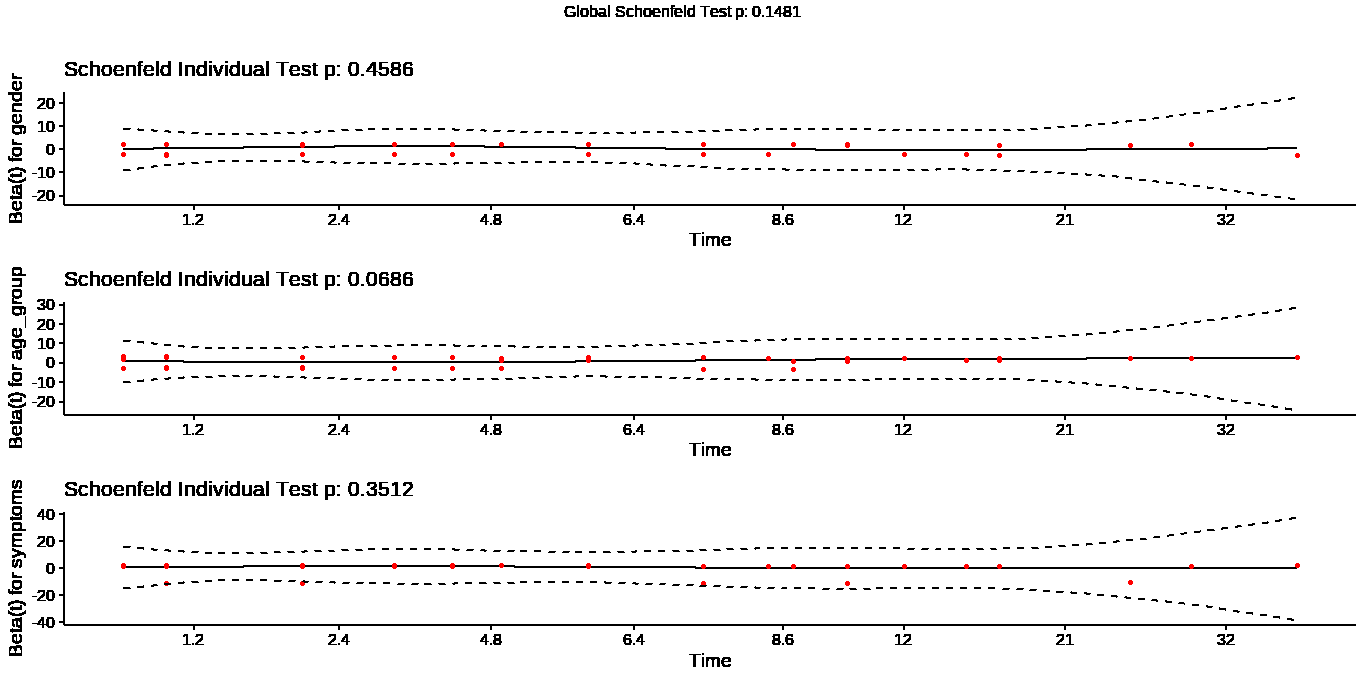


***Supplementary table 2a. Home clinical evaluations performed by SCCU in the five considered epidemic periods (N, peak value of 7-day moving average)***

| **Epidemiological period** | **Analyzed period  (date range)** | **N** | **Peak of 7-day moving average (date)** | **Peak value of 7-day moving average** |
| --- | --- | --- | --- | --- |
| **1st period** | 22/08/2020 – 05/09/2020 | 134 | 29/08/2020 | 11.7 |
| **2nd period** | 30/10/2020 – 13/11/2020 | 812 | 06/11/2020 | 58.6 |
| **3rd period** | 01/04/2021 – 15/04/2021 | 864 | 08/04/2021 | 59.6 |
| **4th period** | 18/08/2021 – 01/09/2021 | 258 | 25/08/2021 | 19.1 |
| **5th period** | 11/01/2022 – 25/01/2022 | 564 | 18/01/2022 | 41.0 |

***Supplementary table 2b. Nasopharyngeal swabs performed by SCCU in the five considered epidemic periods (N, peak value of 7-day moving average)***

| **Epidemiological period** | **Analyzed period  (date range)** | **N** | **Peak of 7-day moving average (date)** | **Peak value of 7-day moving average** |
| --- | --- | --- | --- | --- |
| **1st period** | 22/08/2020 – 05/09/2020 | - | - | - |
| **2nd period** | 01/04/2021 – 15/04/2021 | 622 | 07/11/2020 | 45.4 |
| **3rd period** | 01/04/2021 – 15/04/2021 | 262 | 08/04/2021 | 20.3 |
| **4th period** | 24/08/2021 – 07/09/2021 | 102 | 31/08/2021 | 9.1 |
| **5th period** | 26/12/2021 – 09/01/2022 | 303 | 02/01/2022 | 21.5 |
