# Supplementary material for: The Activity of Special Continuity Care Units in the City of Florence During the COVID-19 Pandemic
Source: Int J Public Health. 2023 Oct 6;68:1606338. doi: 10.3389/ijph.2023.1606338 (PMC10587394; doi:10.3389/ijph.2023.1606338)
Supplement: Supplementary file 2 [file DataSheet1.docx]

**Supplementary File**

*Supplementary table 1a. Home clinical evaluations performed by SCCU in the five considered epidemic periods (N, peak value of 7-day moving average)*

| **Epidemiological period** | **Analyzed period  (date range)** | **N** | **Peak of 7-day moving average (date)** | **Peak value of 7-day moving average** |
| --- | --- | --- | --- | --- |
| **1st period** | 22/08/2020 – 05/09/2020 | 134 | 29/08/2020 | 11.7 |
| **2nd period** | 30/10/2020 – 13/11/2020 | 812 | 06/11/2020 | 58.6 |
| **3rd period** | 01/04/2021 – 15/04/2021 | 864 | 08/04/2021 | 59.6 |
| **4th period** | 18/08/2021 – 01/09/2021 | 258 | 25/08/2021 | 19.1 |
| **5th period** | 11/01/2022 – 25/01/2022 | 564 | 18/01/2022 | 41.0 |

*Supplementary table 1b. Nasopharyngeal swabs performed by SCCU in the five considered epidemic periods (N, peak value of 7-day moving average)*

| **Epidemiological period** | **Analyzed period  (date range)** | **N** | **Peak of 7-day moving average (date)** | **Peak value of 7-day moving average** |
| --- | --- | --- | --- | --- |
| **1st period** | 22/08/2020 – 05/09/2020 | - | - | - |
| **2nd period** | 01/04/2021 – 15/04/2021 | 622 | 07/11/2020 | 45.4 |
| **3rd period** | 01/04/2021 – 15/04/2021 | 262 | 08/04/2021 | 20.3 |
| **4th period** | 24/08/2021 – 07/09/2021 | 102 | 31/08/2021 | 9.1 |
| **5th period** | 26/12/2021 – 09/01/2022 | 303 | 02/01/2022 | 21.5 |
